# Munc18-1 induces conformational changes of syntaxin-1 in multiple intermediates for SNARE assembly

**DOI:** 10.1038/s41598-020-68476-3

**Published:** 2020-07-15

**Authors:** Sanghwa Lee, Jonghyeok Shin, Younghun Jung, Heyjin Son, Jaeil Shin, Cherlhyun Jeong, Dae-Hyuk Kweon, Yeon-Kyun Shin

**Affiliations:** 1 Advanced Photonics Research Institute, Gwangju Institute of Science and Technology, Gwangju, 61005 Republic of Korea; 2 Department of Integrative Biotechnology, College of Biotechnology and Bioengineering, Sungkyunkwan University, Suwon, 16419 Republic of Korea; 3 Department of Biochemistry Biophysics and Molecular Biology, Iowa State University, Ames, IA 50011 USA; 4 Center for Theragnosis, Korea Institute of Science and Technology, Seoul, 02792 Republic of Korea; 5 KHU-KIST Department of Converging Science and Technology, Kyunghee University, Seoul, 02447 Republic of Korea; 6 Present Address: Carl R. Woese Institute for Genomic Biology, University of Illinois at Urbana-Champaign, Urbana, IL 61801 USA; 7 Present Address: Institute of BioInnovation Research, Kolon Life Science, Seoul, 07793 Republic of Korea

**Keywords:** Single-molecule biophysics, Membrane structure and assembly, Exocytosis, Membrane proteins

## Abstract

In neuronal exocytosis, SNARE assembly into a stable four-helix bundle drives membrane fusion. Previous studies have revealed that the SM protein Munc18-1 plays a critical role for precise SNARE assembly with the help of Munc13-1, but the underlying mechanism remains unclear. Here, we used single-molecule FRET assays with a nanodisc membrane reconstitution system to investigate the conformational dynamics of SNARE/Munc18-1 complexes in multiple intermediate steps towards the SNARE complex. We found that single Munc18-1 proteins induce the closed conformation of syntaxin-1 not only in the free syntaxin-1 but also in the t-SNARE (syntaxin-1/SNAP-25) complex. These results implicate that Munc18-1 may act as a gatekeeper for both binary and ternary SNARE complex formation by locking the syntaxin-1 in a cleft of Munc18-1. Furthermore, the kinetic analysis of the opening/closing transition reveals that the closed syntaxin-1 in the syntaxin-1/SNAP-25/Munc18-1 complex is less stable than that in the closed syntaxin-1/Munc18-1 complex, which is manifested by the infrequent closing transition, indicating that the conformational equilibrium of the ternary complex is biased toward the open conformation of syntaxin-1 compared with the binary complex.

## Introduction

Neuronal exocytosis for neurotransmitter release is driven by the assembly of the three soluble *N*-ethylmaleimide-sensitive factor attachment protein receptor (SNARE) proteins, which are syntaxin-1 and SNAP-25 in the presynaptic plasma membrane and synaptobrevin in the synaptic vesicle membrane^1,2^. The assembly of these proteins generates a stable four-helix bundle between the vesicle and target membranes, thus promoting membrane fusion^3,4^. Although the membrane fusion can be induced by the SNAREs alone in vitro, a number of auxiliary proteins are required for membrane fusion with high speed and high fidelity in vivo^5,6^. Among those, the Sec1/Munc18 (SM) family proteins Munc18-1 and Munc13-1 are known to be essential for SNARE-mediated membrane fusion^7–9^.

Extensive studies on roles of the Munc18-1 and Munc13-1 in the membrane fusion have established that these proteins are critically involved in the regulation of SNARE complex formation. Initially, Munc18-1 induces the closed conformation of syntaxin-1, locking the syntaxin-1 protein in a cleft of Munc18-1, that inhibits the spontaneous binding of SNAP-25 to syntaxin-1^10,11^. Recent reports have revealed that the MUN domain of Munc13-1 promotes the transition from the syntaxin-1/Munc18-1 complex to the ternary SNARE complex in the presence of SNAP-25 and synaptobrevin, suggesting that Munc13-1 plays a role in opening syntaxin-1 for the subsequent SNARE assembly^12–15^. On the other hand, it is also known that Munc18-1 stimulates membrane fusion when it binds to a fully assembled SNARE complex^16–18^.

Despite such major advances, many important questions concerning the mechanisms underlying the precise regulation of SNARE complex formation by the Munc18-1 and Munc13-1 still remain unanswered. For instance, the kinetic pathway of SNARE assembly has been extensively studied, but the sequential order of binding of the other SNARE partners (SNAP-25 and synaptobrevin) has not yet been clarified^13,19–25^. Furthermore, the structural and dynamic features of intermediates remain elusive.

Here, we perform a single-molecule fluorescence resonance energy transfer (FRET) assay with a nanodisc membrane reconstitution system^26,27^, which allows us to observe conformational dynamics of syntaxin-1 in the intermediate stages of SNARE assembly in the presence and absence of Munc18-1. In this unique experiment, we focused on the mechanism underlying the regulation of SNARE assembly by Munc18-1 in the intermediate states. Our results strongly suggest that Munc18-1 acts as a chaperone not only in the syntaxin-1 alone but also in the t-SNARE complex (syntaxin-1/SNAP-25 complex) by dynamically inducing and modulating the closed conformation of syntaxin-1.

## Results

### Single-molecule FRET assay for observing intermediate states during t-SNARE complex formation

**Figure 1:**
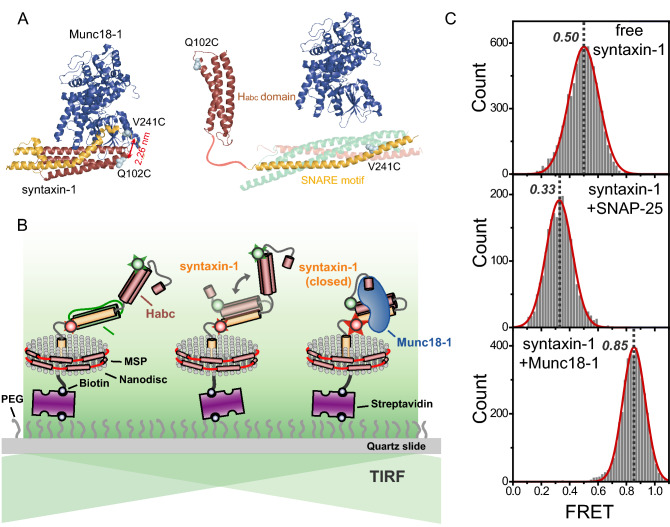
Single-molecule FRET measurement of syntaxin-1 conformations in t-SNARE complex formation. (**A**) Structures of the Munc18-1/syntaxin-1 complex (Protein Data Bank code 3C98) and the SNAREs (Protein Data Bank codes 1SFC for SNARE complex and 1BR0 for H_abc_ domain). The two cysteine mutations, Q102C and V241C, introduced for site-specific dye labeling are indicated. The distance between the Cα atoms of these residues in the Munc18-1/syntaxin-1 complex was estimated to be 2.26-nm. (**B**) Schematic diagram of single-molecule FRET assay with membrane reconstitution of syntaxin-1. (**C**) FRET histograms for syntaxin-1 proteins (free syntaxin-1 only, top; with bound SNAP-25, middle; with bound Munc18-1, bottom). FRET values for each construct were obtained by fitting the FRET histograms to a single Gaussian function. To obtain the FRET histograms, all data points for each construct were collected from at least more than 50 time trajectories.

To investigate the conformational dynamics of syntaxin-1 during t-SNARE complex formation, we first labeled syntaxin-1 with a FRET dye pair, the fluorescence donor Cy3 at the H_abc_ domain and the acceptor Cy5 at the SNARE motif of syntaxin-1. We selected the labeling positions Val241 and Gln102 such that substantial FRET changes arise during the transitions between the open and closed conformations of syntaxin-1 as depicted in Fig. 1A. Using the SDS resistance assay, we confirmed that cysteine mutations does not negate the SDS-resistant property of the SNARE complex (Supplementary Fig. S1). For membrane-reconstitution of syntaxin-1, we employed a nano-sized lipid bilayer called a nanodisc and reconsitituted a single syntaxin-1 protein into a nanodisc (see “Methods” section). To observe the conformational changes in syntaxin-1 in real time, we immobilized the nanodisc containing a doubly-labeled syntaxin-1 on a polyethylene glycol (PEG)-coated imaging surface using biotinylated lipids of the nanodiscs via a streptavidin–biotin interaction and monitored fluorescence signals from single syntaxin-1 molecules individually using a total internal reflection microscope (Fig. 1B).

As the previous single-molecule multiparameter fluorescence detection (SmMFD) experiment revealed^28^, free syntaxin-1 is expected to be in a dynamic equilibrium between the closed and open conformations. In our measurement with free syntaxin-1, however, we were not able to detect such fast transitions between two states in a submilllisecond time scale due to the limited time resolution (Fig. 1C, top). When bound to SNAP-25, however, syntaxin-1 exhibited a much lower FRET distribution than free syntaxin-1 (Fig. 1C, middle), indicating that the binding of SNAP-25 to syntaxin-1opens up the syntaxin-1 conformation. On the other hand, in the presence of bound Munc18-1, we obtained a higher FRET distribution (Fig. 1C, bottom) than that of free syntaxin-1, indicating that Munc18-1 arrests the closed conformation of syntaxin-1. Putting together, although the transitions between the closed and open states in free syntaxin-1 were not directly accessible, most likely due to the fast equilibrium, we could infer the dynamic equilibrium between the two states via FRET peak shift. The mean value of the two FRET peaks corresponding to the closed and open conformations, which obtained from FRET histograms for SNAP-25 and Munc18-bound syntaxin-1 proteins respectively, was similar to the observed FRET value for free syntaxin-1. Collectively, based on these observations, we could clearly assign the observed FRET values to intermediate states during t-SNARE complex formation in the presence of Munc18-1.

### Dynamic transitions between the closed syntaxin-1/Munc18-1 complex and free syntaxin-1

**Figure 2:**
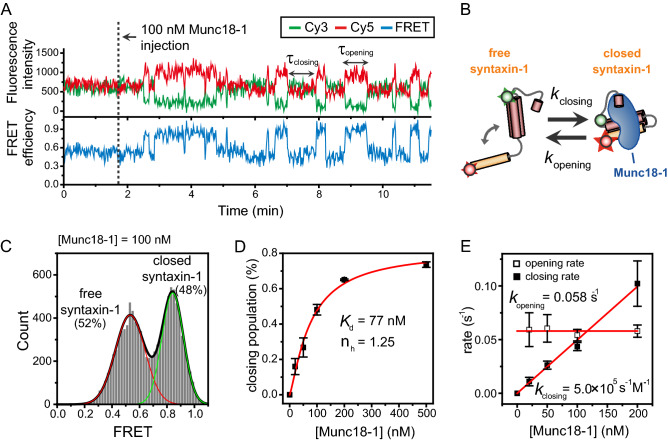
Kinetic analysis of the closing and opening of syntaxin-1 by Munc18-1. (**A**) Representative time traces of fluorescence intensity and FRET efficiency showing the closing and opening of syntaxin-1 by Munc18-1. Cy3 fluorescence, Cy5 fluorescence, and corresponding FRET efficiency are shown in green, red, and blue, respectively. This same color convention is used throughout the paper. τ_closing_ (or dwell time of open syntaxin-1) indicates the time for syntaxin-1 to undergo a conformational change from the open to the closed state, while τ_opening_ (or dwell time of closed syntaxin-1) indicates the time for the opposite case. (**B**) Kinetic scheme for the Munc18-induced syntaxin-1 closing/opening reaction. (**C**) FRET histograms of syntaxin-1 in the presence of 100 nM Munc18-1. Relative populations for each state were obtained by fitting the FRET histogram to sum of two Gaussian functions. (**D**) Relative population of the closed syntaxin-1 conformation at varying Munc18-1 concentrations. The dissociation constant and hill coefficient of the Munc18-1 were obtained from a hyperbolic Hill fit of the data. (**E**) Closing and opening rates at varying Munc18-1 concentrations. The closing rate constant (5.0 × 10^5^ s^−1^ M^−1^) was obtained from a linear fit of the data. The average of opening rates is 0.058 s^−1^. Error bars in (**D**, **E**) represent standard deviations obtained from two independent experiments.

By using the unique capability of our single-molecule FRET assay to identify individual intermediates, we first investigated dynamic interactions between syntaxin-1 and Munc18-1 in real time. Representative time traces of the fluorescence intensity and the FRET efficiency from a real-time Munc18-1 injection experiment is shown in Fig. 2A. In this experiment, Munc18-1 proteins were delivered into the nanodisc-immobilized flow chamber while fluorescence signals from single syntaxin-1 molecules in the nanodisc were being monitored. Before the Munc18-1 injection, fluorescence intensities showed a single-state behavior with middle FRET value (~ 0.50), which indicates the fast dynamic equilibrium between the open and closed states for free syntaxin-1 as described in Fig. 1C. Upon the addition of Munc18-1, however, we observed clear two-state dynamics between a middle FRET state for free syntaxin-1 and a high FRET state (~ 0.85) for the closed syntaxin-1/Munc18-1 complex (Fig. 2A, B). The FRET histogram also established the existence of the two distinct conformational states in the presence of Munc18-1 (Fig. 2C). These observations are in line with the previous crystal structure of the closed syntaxin-1/Munc18-1 complex^10^.

To examine the conformational dynamics of syntaxin-1 in connection with Munc18-1in further details, we performed Munc18-1 titration experiments (Supplementary Fig. S2). As shown in Fig. 2D, we determined the dissociation constant and the Hill coefficient on the basis of relative populations of the closed syntaxin-1/Munc18-1 complex as a function of Munc18-1 concentration. Likely due to steric hindrance by the dye labeling of syntaxin-1, the dissociation constant showed a considerable reduction in the binding affinity of Munc18-1 compared to the values obtained by other methods for unlabeled syntaxin-1^10,29^. Nonetheless, the Hill plot analysis revealed a Hill coefficient close to 1, which suggests the 1:1 stoichiometric interaction of Munc18-1 with closed syntaxin-1. This conclusion is further supported by the kinetic analysis of the closing (or binding) and opening (or unbinding) transitions for syntaxin-1 at varying Munc18-1 concentrations (Supplementary Fig. S3). The analysis of the closing and opening kinetics revealed that the closing rate increased linearly with the increase of the Munc18-1 concentration, while the opening rates remained almost constant over the protein concentration range examined (Fig. 2E). Thus, the results suggest that the closed conformation of a single syntaxin-1 is induced by the binding of a single Munc18-1.

### Munc18-1 dynamically induces the closed syntaxin-1 conformation even in the t-SNARE complex

**Figure 3:**
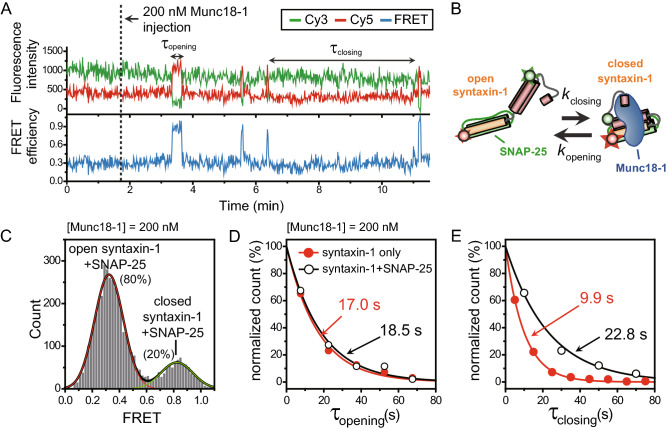
Direct observation of Munc18-induced the closed syntaxin-1 conformation in t-SNARE complex. (**A**) Representative time traces of fluorescence intensity and FRET efficiency showing the closing and opening of syntaxin-1 by Munc18-1 in the presence of bound SNAP-25. τ_closing_ (or dwell time of open syntaxin-1) indicates the time for syntaxin-1 to undergo a conformational change from the open to the closed state, while τ_opening_ (or dwell time of closed syntaxin-1) indicates the time for the opposite case. (**B**) Kinetic scheme for the Munc18-induced syntaxin-1 closing/opening reaction in t-SNARE complex. (**C**) FRET histograms of syntaxin-1 with bound SNAP-25 and 200 nM Munc18-1. Relative populations for each state were obtained by fitting the FRET histogram to sum of two Gaussian functions. (**D**) Dwell time histograms of closed syntaxin-1 with (open circles) and without (red solid circles) bound SNAP-25. (**E**) Dwell time histograms of open syntaxin-1 with (open circles) and without (red solid circles) bound SNAP-25. In (**C**, **D**), the data were fit to single-exponential decay functions (solid lines) to obtain each average dwell time.

Recent studies strongly suggested that Munc18-1 may act as a regulator for multiple sequential steps during SNARE assembly^19–22,24,25,30^. However, the dynamic nature of the regulation of the intermediate steps by Munc18-1 is not fully understood. As an initial step toward the understanding of the regulation by Munc18-1, we investigated if Munc18-1 forms a ternary complex with SNAP-25 and syntaxin-1 and induces the closed conformation of syntaxin-1 to possibly prevent the syntaxin-1 from the unregulated binding of the vesicle SNARE (v-SNARE) synaptobrevin. We thus reconstituted syntaxin-1/SNAP-25 complex into a nanodisc and immobilized the nanodisc on the PEG-coated imaging surface for single-molecule FRET measurements. Figure 3A shows the representative time traces of syntaxin-1 in the complex with SNAP-25 from the real-time Munc18-1 injection experiment. Strikingly, upon the addition of Munc18-1, we observed clear two state dynamics between a low FRET state (~ 0.33) that corresponds to the open syntaxin-1 state and a short-lived, but clear high FRET state representing closed syntaxin-1 (Fig. 3A, B). We did not observe any middle FRET state (~ 0.5) reflecting free syntaxin-1, indicating that the observed transitions are not likely due to the dissociation of SNAP-25 from the complex. In this measurement, the existence of transitions from low to high FRET states can be also interpreted as displacement of SNAP-25 from syntaxin-1 by Munc18-1. However, in Fig. 3A, we found that syntaxin-1 exhibited low FRET states, which represent SNAP-25 bound states, whenever Munc18-1 dissociated from the closed complex. By combining this result with the fact that unbound SNAP-25 can rarely recombine with syntaxin-1 because there is a very small amount of SNAP-25 in the detection chamber even if most of SNAP-25 proteins is displaced from syntaxin-1 by Munc18-1, we excluded the possibility regarding the displacement of SNAP-25 by Munc18-1. Therefore, we concluded that Munc18-1 could arrest the closed conformation of syntaxin-1 even in the syntaxin-1/SNAP-25 complex, even if SNAP-25 does not dissociate from the ternary complex. FRET histogram (Fig. 3C) and kinetic analysis (Fig. 3D, E) of these transitions revealed that Munc18-1 showed a significant reduction in the ability to arrest the closed conformation of syntaxin-1 in the presence of bound SNAP-25 compared to that of free syntaxin-1. In addition, it is notable that the frequency of the closing transitions (1/τ_closing_) in the presence of bound SNAP-25 decreased significantly, while the dwell time of the closed state (τ_opening_) did not show any appreciable changes compared to that of free syntaxin-1. The result indicates that the infrequent closing events are primarily responsible for the decrease of the high FRET population in the presence of bound SNAP-25 (Fig. 3C).

## Discussion

**Figure 4:**
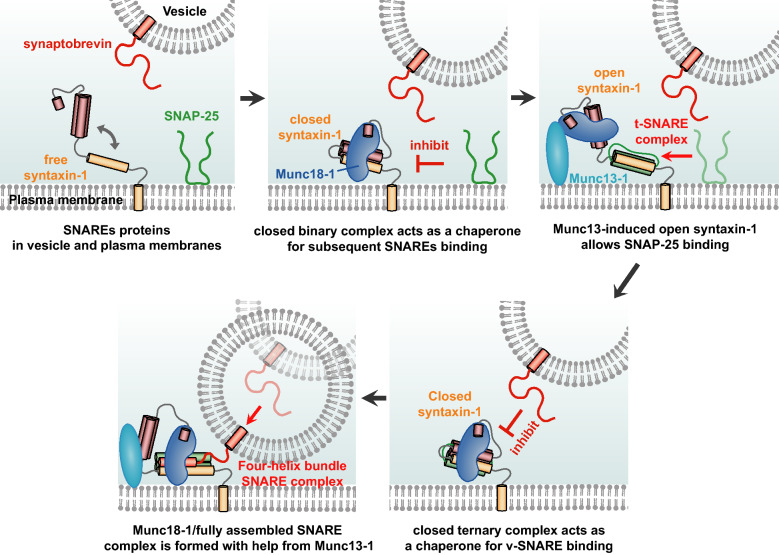
Proposed model for the regulation of SNARE assembly by Munc18-1. Based on our observations, we propose a model of sequential Munc18-1 operation during the SNARE assembly process. Munc18-1 first binds to syntaxin-1 with a 1:1 molecular interaction and then induces the closed conformation in syntaxin-1. This closed binary complex acts as a chaperone for the other SNAREs binding by locking the syntaxin-1. When Munc13-1 opens syntaxin-1, SNAP-25 binds to syntaxin-1 and thus the t-SNARE complex is formed with Munc18-1. In this intermediate, the closed ternary complex also acts as a chaperone for the v-SNARE synaptobrevin binding by locking the syntaxin-1 in a cleft of Munc18-1. When the ternary complex is opened by Munc13-1, a fully assembled SNARE complex is formed and then Munc18-1 catalyzes membrane fusion.

Identification and characterization of key intermediates along the pathway of SNARE assembly has long been a topic of intense interest. Due to the transient nature of these intermediates, however, a deeper understanding of these intermediate states has been hindered by population averaging that is inherent in ensemble measurements. Therefore, we have developed the single-molecule FRET assay with nanodiscs, which was capable of monitoring conformational dynamics of the key intermediates. This method enables the structural and dynamic characterization of the intermediates, which is summarized in Fig. 4 and below.

Previously, it has long been known that, at the early stage of SNARE assembly, Munc18-1 forms the closed syntaxin-1/Munc18-1 complex and this complex plays a role in the inhibition of the subsequent steps in SNARE assembly^10,11,31^. Consistently, our results show that Munc18-1 dynamically induces the closed conformation in the syntaxin-1. Our kinetic analysis of the conformational dynamics additionally revealed that the closed syntaxin-1/Munc18-1 complexes are dynamically induced by 1:1 stoichiometric interaction between single syntaxin-1 and Munc18-1 proteins.

This closed syntaxin-1/Munc18-1 complex at the early stage of SNARE assembly has been well characterized previously, but there has been still controversy regarding the subsequent steps. Previous extensive studies have suggested two alternative models for kinetic pathway of subsequent assembly. In the first model, Munc18-1 forms a ternary complex with syntaxin-1 and SNAP-25, which may be a key intermediate for the subsequent binding of synaptobrevin^17,21–23^. In contrast, the second model suggests that the subsequent intermediate is a template complex in which Munc18-1 arranges initially syntaxin-1 and synaptobrevin in a cleft of Munc18-1^20,24,25,30^. In this study, we found that Munc18-1 could bind to t-SNARE complex and induce the closed conformation of syntaxin-1 similar to the early stage even in this ternary complex, strongly supporting the first model for the kinetic pathway of SNARE assembly. This finding implies that the ternary complex may act as a critical intermediate state for the subsequent synaptobrevin binding and, in this step, Munc18-1 may also play a role as chaperone for subsequent steps by locking the syntaxin-1 in a cleft of Munc18-1.

The existence of the closed syntaxin-1 conformation in the ternary complex raises the question regarding the kinetic difference between two critical intermediates, syntaxin-1/Munc18-1 and syntaxin-1/SNAP-25/Munc18-1 complexes. Our kinetic analysis of the open/closing dynamics of syntaxin-1 in these two intermediates revealed that the closed ternary syntaxin-1/SNAP-25/Munc18-1 complex is less stable than the closed binary syntaxin-1/Munc18-1 complex, which is apparent in the infrequent closing transitions of the ternary complex. Consistent with this observation, a recent electron paramagnetic resonance spectroscopy study showed that the binding of SNAP-25 to the binary syntaxin-1/Munc18-1 complex biased the conformational equilibrium toward the open conformation of syntaxin-1^21^.

In conclusion, although it remains to be unambiguously proven, our data strongly supports the model that Munc18-1 acts as a functional template and chaperone to promote precise SNARE assembly in multiple intermediates during SNARE assembly with help from Munc13-1^32^.

## Methods

### Protein expression and purification

Wild-type syntaxin-1A, cysteine mutant of syntaxin-1A, wild-type SNAP-25, wild-type synaptobrevin-2, ApoA1 were expressed and purified based on the previous studies^26,33^. Plasmid harboring each protein was introduced in *E. coli* BL21 (DE3) Rosetta pLysS (Novagen). Cells were cultured in Luria–Bertani medium and induced to express protein in early-exponential phase at 16 °C overnight. Cells were harvested and resuspended with PBS. Cells were lysed with sonication. GST fused proteins were purified using glutathione-agarose beads (Thermo Scientific). Proteins were eluted with cleavage of thrombin in thrombin cleavage buffer (50 mM Tris, 150 mM NaCl, 2.5 mM CaCl2, 2 mM TCEP, pH 8.0) containing 1% *n*-octyl glucoside in case of membrane protein.

Munc18 was expressed and purified based on the previous study^23^. Plasmid harboring the Munc18 was introduced in BL21 (DE3) Rosetta pLysS (Novagen). Cells were cultured in the same manner as mentioned above, protein expression was induced at 20 °C. Cells were harvested and resuspended with lysis buffer (25 mM HEPES, 100 mM KCl, 20 mM imidazole, 2 mM AEBSF, 2 mM DTT, pH 7.4). Cells were lysed with sonication. The supernatant of lysate was incubated with Ni–NTA bead (Qiagen). Beads were washed with lysis buffer and eluted with elution buffer (25 mM HEPES, 100 mM KCl, 100 mM imidazole, 2 mM AEBSF, 2 mM DTT, pH 7.4).

For dye labeling, syntaxin-1A cysteine mutant was constructed by introducing two mutations (V241C and Q102C), using site-directed mutagenesis. The purified syntaxin-1A mutant should be stored in thrombin cleavage buffer with 1% *n*-octyl glucoside. Protein, donor dye, and acceptor dye were mixed at a molar ratio of 1:10:20. The mixture of protein and dye was incubated at 4 °C in the dark with gentle shaking overnight. Free dyes were removed by fractionating with PD MiniTrap G-25 desalting column.

### Preparation of syntaxin-1 and t-SNARE reconstituted nanodiscs

Reconstitution of the nano-sized lipid bilayer, nanodisc bearing purified recombinant syntaxin-1A (hereafter syntaxin-1) or t-SNARE complex was performed as described previously^26^. First, phospholipid mixture was prepared by mixing 1-palmitoyl-2-oleoyl-*sn*-glycero-3-phosphocholine (POPC), 1,2-dioleoyl-*sn*-glycero-3-phospho-l-serine (DOPS), and 16:0 Biotinyl Cap PE, 1,2-dipalmitoyl-sn-glcero-3-phosphoethanolamine-N (Biotin-DPPE) (Avanti Polar Lipids) to a molar ratio of 85:15:0.5 at a final concentration of 50 mM. Dried lipid mixtures were resuspended with PBS and stored at − 80 °C deep freezer. Then, phospholipid mixture, sodium cholate, labeled syntaxin-1 (or labeled t-SNARE complex), and ApoA1 protein (membrane scaffold protein) were mixed to a molar ratio 300:5:0.25:1 and incubated on the ice for 10 min. To generate the binary complex of syntaxin-1A and SNAP-25 (t-SNARE complex), purified syntaxin-1A and SNAP-25 with a molar ratio of 1:1 were pre-incubated at 4 °C for 1 h. The same volume of bio-bead solution was added to induce self-assembly of nanodisc by removing sodium cholate. Mixtures were incubated at 4 °C with gentle shaking. The syntaxin-1 (or t-SNARE complex) reconstituted nanodisc was purified using FPLC equipped with Superdex™ 200 GL 10/30 column (Amersham Bioscience).

### Single-molecule FRET experiments

To prevent the non-specific adsorption of nanodiscs and proteins to the surface, cleaned quartz microscope slides and coverslips were coated with polyethylene glycol (m-PEG-5000; Laysan Bio Inc.) and biotinylated polyethylene glycol (biotin-PEG-5000; Laysan Bio, Inc.) in a 40:1 ratio^34^. A sample chamber was constructed between a microscope slide and a coverslip using double-sided adhesive tape. Nanodiscs containing biotinylated lipids were immobilized on the PEG-coated surface via a streptavidin–biotin interaction. Using a home-built prism-type total internal reflection fluorescence microscope, single-molecule fluorescence images were taken in an imaging buffer (10 mM Tris–HCl (pH 8.0) with 0.4% (w/v) glucose (Sigma, USA), 1% (v/v) Trolox (Sigma, USA), 1 mg/mL glucose oxidase (Sigma, USA), 0.04 mg/mL catalase (Sigma, USA), 100 mM KCl, Munc18-1 with varying concentration) to reduce photobleaching and blinking of dyes^35^. During the buffer injection experiments, a new buffer containing Munc18-1 proteins was infused into the detection chamber by using a syringe pump (Fusion 200; Chemyx) while single-molecule fluorescence images from doubly labeled syntaxin-1 were being taken. As an excitation source for two-color FRET, a green laser (532 nm, Sapphire; Coherent) was used. Fluorescence signals from Cy3 and Cy5 were collected using a water immersion objective lens (UPlanSApo 60x; Olympus), filtered by a 532 nm long-pass filter (LP03-532RU-25; Semrock), separated through a dichroic mirror (635dcxr; Chroma), and imaged onto an electron multiplying (EM)-charge coupled device (CCD) camera (Ixon DU897D; Andor).

### Analysis of single-molecule FRET data

To collect only doubly labeled syntaxin-1 from heterogeneous populations, a few hundred single-molecule spots from the images obtained an acceptor detection channel were selected following 532 nm laser excitation. The time courses of Cy3 and Cy5 signals from a single molecule were extracted from a recorded movie file using IDL (ITT Visual Information Solutions) and analyzed using a custom program written in MATLAB (Mathwork) scripts. To calculate FRET efficiency, which is defined as the ratio of acceptor intensity to the sum of donor and acceptor intensities, data collection including background subtraction and cross-talk correction of the donor signal to the acceptor detection channel was performed. For kinetic analysis of the time traces, FRET states were determined by the standard threshold method using FRET time traces. To obtain the kinetic times, dwell time histograms for each FRET state were fitted by an exponential decay function. Then, the transition rates were determined by inverting the average dwell times. In general, kinetic rates of stochastic transitions in a two-state system can be determined from the average dwell times for two conformational states^34^.

## Supplementary information

Supplementary information.

## Data Availability

All data are available upon request to the corresponding authors.
