# Supplementary material for: Munc18-1 induces conformational changes of syntaxin-1 in multiple intermediates for SNARE assembly
Source: Sci Rep. 2020 Jul 15;10:11623. doi: 10.1038/s41598-020-68476-3 (PMC7363831; doi:10.1038/s41598-020-68476-3)
Supplement: Supplementary file 1 — Supplementary information. [file 41598_2020_68476_MOESM1_ESM.docx]

**Supplementary Information**

**Munc18-1 induces conformational changes of syntaxin-1 in multiple intermediates for SNARE assembly**

Sanghwa Lee^1,6,*^, Jonghyeok Shin^2,6,7^, Younghun Jung^2^, Heyjin Son^1^, Jaeil Shin^3,8^, Cherlhyun Jeong^4,5^, Dae-Hyuk Kweon^2^, and Yeon-Kyun Shin^3,*^

^1^Advanced Photonics Research Institute, Gwangju Institute of Science and Technology, Gwangju, 61005, Republic of Korea. ^2^Department of Integrative Biotechnology, College of Biotechnology and Bioengineering, Sungkyunkwan University, Suwon, 16419, Republic of Korea. ^3^Department of Biochemistry Biophysics and Molecular Biology, Iowa State University, Ames, IA, 50011, USA. ^4^Center for Theragnosis, Korea Institute of Science and Technology, Seoul, 02792, Republic of Korea. ^5^KHU-KIST Department of Converging Science and Technology, Kyunghee University, Seoul, 02447, Republic of Korea.

^6^These authors contributed equally to this work.

^7^Present address: Carl R. Woese Institute for Genomic Biology, University of Illinois at Urbana-Champaign, Urbana, IL, 61801, USA.

^8^Present address: Institute of BioInnovation Research, Kolon Life Science, Seoul, 07793, Republic of Korea.

^*^Correspondence and requests for materials should be addressed to Sanghwa Lee (email: sanglee@gist.ac.kr) or Yeon-Kyun Shin (email: colishin@iastate.edu)

*Table of Contents:*

*Figure S1.* SDS-resistant SNARE complex formation of wild-type and cysteine mutant syntaxin-1

*Figure S2.* Munc18-1 titration experiments

*Figure S3.* Analysis of average dwell times for open and closed syntaxin-1 in the presence of Munc18-1

**Figure S1.** SDS-resistant SNARE complex formation of wild-type and cysteine mutant syntaxin-1


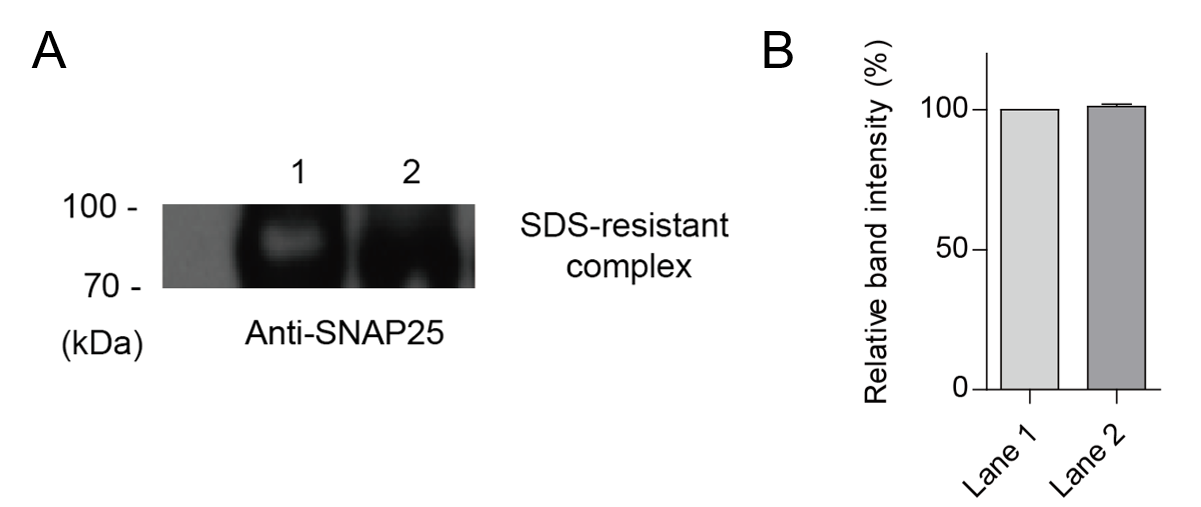


(A) Same amounts of purified SNARE proteins were mixed and incubated in 37 ^o^C for 1 hour. Unboiled mixtures were loaded on the SDS-PAGE. SDS-resistant SNARE complex was identified by western blot using SNAP-25 antibody. Lane 1, mixture of wild-type syntaxin-1, wild-type synaptobrevin-2, and wild-type SNAP-25; Lane 2, mixture of syntaxin-1 cysteine mutant (Q102C/V241C), wild-type synaptobrevin-2, and wild-type SNAP-25. (B) Relative band intensity of SDS-resistant complex. Results are the means of the experimental data from biological replicates (n=2). The error bar represents the standard deviation.

**Figure S2.** Munc18-1 titration experiments


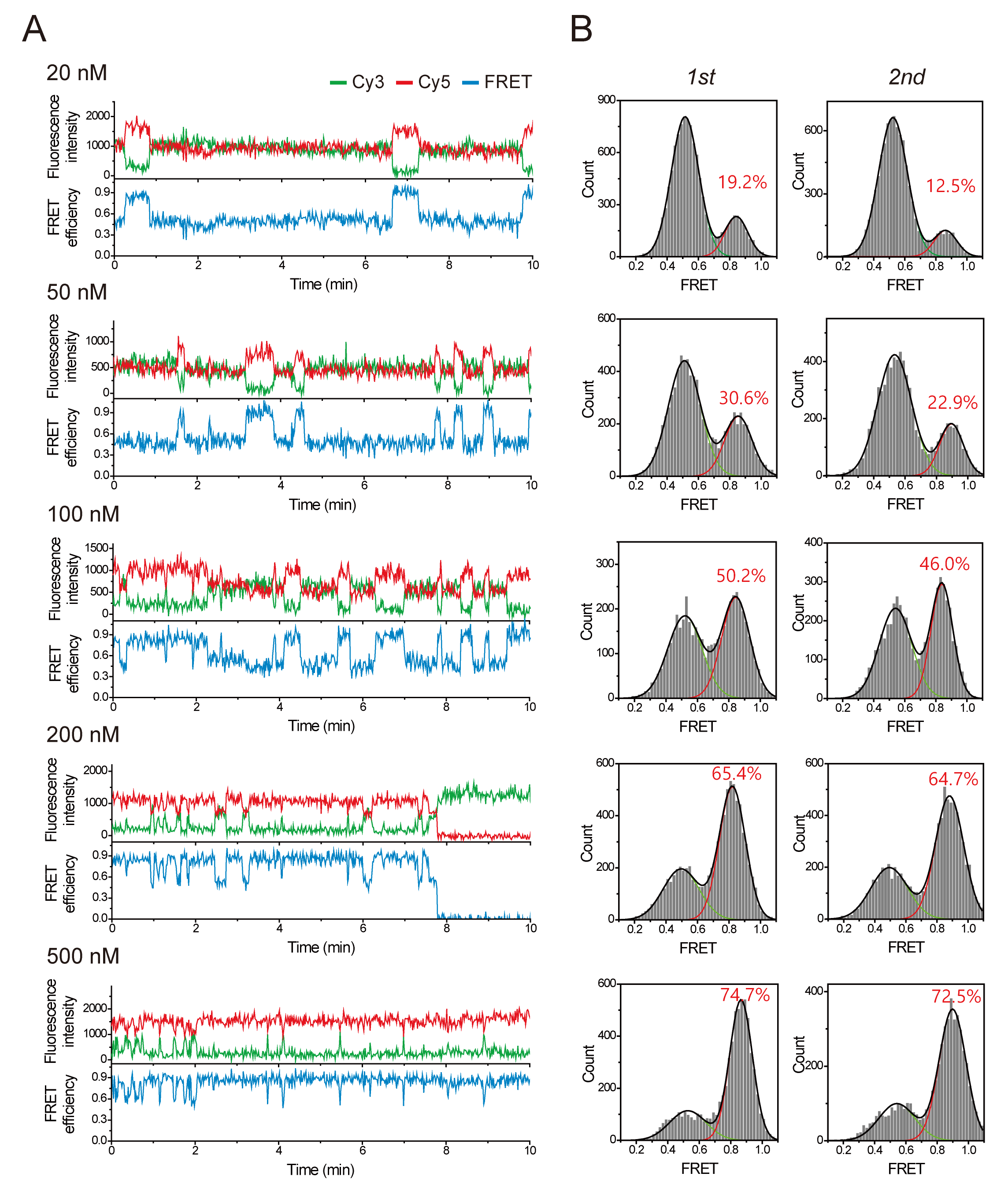


(A) Representative fluorescence intensity (shown in green for Cy3 fluorescence; in red for Cy5 fluorescence) and FRET (shown in blue for FRET efficiency) time traces showing the closing and opening of syntaxin-1 at varying Munc18-1 concentrations. (B) Corresponding FRET histograms of syntaxin-1. Relative populations of the closed syntaxin-1 were obtained by fitting the FRET histograms to sum of two Gaussian functions. To obtain error bars in Fig. 2D, each experiment was repeated two times. All FRET histograms were obtained by analyzing at least 30 molecules.

**Figure S3.** Analysis of average dwell times for open and closed syntaxin-1 in the presence of Munc18-1


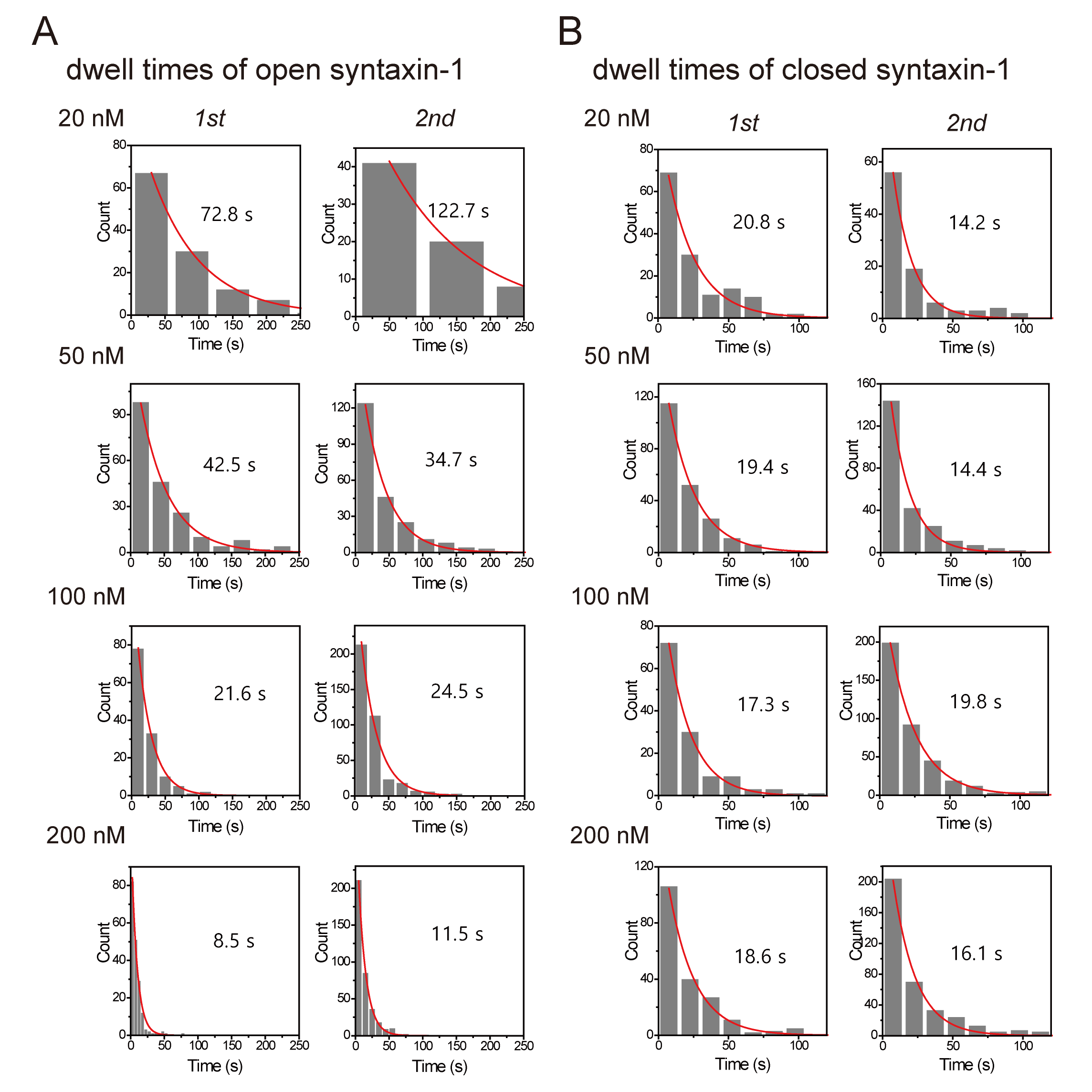


(A and B) Dwell time histograms of open (A) and closed (B) syntaxin-1 at varying Munc18-1 concentrations. To obtain error bars in Fig. 2E, each experiment was repeated two times. Each histogram was fit by a single-exponential decay function to obtain the corresponding kinetic time.
